# Skin Shade Discrimination Is Associated With Disordered Weight Control Behaviors in Hong Kong, Malaysia, Singapore, and Thailand

**DOI:** 10.1002/eat.70117

**Published:** 2026-04-30

**Authors:** Nadia Craddock, S. Bryn Austin, Flora Or, Wipada Rodtanaporn, Paul White, Sook Ning Chua

**Affiliations:** ^1^ Centre for Appearance Research, University of the West of England Bristol UK; ^2^ Department of Social and Behavioral Sciences Harvard T.H. Chan School of Public Health Boston Massachusetts USA; ^3^ Division of Adolescent and Young Adult Medicine Boston Children's Hospital Boston Massachusetts USA; ^4^ Relate Mental Health Malaysia Kuala Lumpur Malaysia

## Abstract

**Objective:**

Emerging evidence indicates that experiencing discrimination is associated with disordered eating. This study aimed to test the association between experiences of colorism (skin shade discrimination penalizing those with darker skin) and the prevalence of disordered weight control behaviors (DWCBs) in four Asian countries/regions.

**Method:**

A total of 5251 adults (52.4% women) from Hong Kong (*n* = 1014), Malaysia (*n* = 1141), Singapore (*n* = 1049), and Thailand (*n* = 2047) completed an online survey. Participants provided demographic information, completed a scale on skin shade discrimination and reported engagement in any DWCBs (e.g., vomiting, fasting, excessive exercise, as well as the use of laxatives, diet pills, fat burners, detox/cleansing dietary supplements, muscle building/sport‐performance dietary supplements, anabolic steroids) in the past 3 months. Multivariable logistic analyses were conducted to estimate the cross‐sectional association between experiences of skin shade discrimination and DWCBs, controlling for demographic information including skin shade, age, gender, and geographic region.

**Results:**

One in four (25.3%) participants reported experiencing skin shade discrimination (ranging from 14.2% in Hong Kong to 31.0% in Thailand) and almost half (53.8%) reported engaging in at least one DWCB in the past 3 months. Overall, experiences of skin shade discrimination were associated with nearly fivefold higher odds of engaging in DWCBs in the same period (5.44; 95% CI [4.67, 6.33]).

**Discussion:**

Findings highlight colorism as a potential sociocultural correlate of DWCBs among adults in Hong Kong, Malaysia, Singapore, and Thailand. This association warrants further investigation.

## Summary

- Experiences of skin shade discrimination were associated with a greater likelihood of engaging in disordered weight control behaviors among adults in Hong Kong, Malaysia, Singapore, and Thailand.
- Individuals reporting recent experiences of skin shade discrimination were substantially more likely to engage in behaviors such as fasting, vomiting, or use of weight‐control products compared with those reporting no discrimination.
- A greater range of skin shade discrimination experiences was associated with increased risk, suggesting a potential cumulative relationship between discrimination and disordered eating behaviors.
- Findings highlight colorism as a potentially important and under‐recognized sociocultural risk factor for disordered eating in East and Southeast Asia, warranting further longitudinal and qualitative research.

## 1 Introduction

Eating disorders are serious, costly illnesses associated with increased disability‐adjusted life years and mortality (Santomauro et al. 2021). Although under‐researched outside high‐income English‐speaking countries, eating disorders are increasingly common across Asia (Kim et al. 2021; Wu et al. 2020). Wu et al. (2020) reported that the largest global increase in eating disorder prevalence between 1990 and 2017 occurred in East Asia. In Southeast Asia, recent studies found 58.5% of adults in Malaysia (Chua et al. 2022) and 42.7% of adults in Singapore (Chua et al. 2021) reported eating disorder symptoms. Therefore, greater insight into risk factors and manifestations of eating disorder symptoms in East and Southeast Asia is needed to guide region‐specific prevention.

Consistent with research linking perceived discrimination to risky health behaviors and adverse wellbeing (Pascoe and Smart Richman 2009; Schmitt et al. 2014), some studies have documented an association between perceived discrimination and eating disorder symptoms (Mason et al. 2021). Mason et al. (2021) suggested that experiencing discrimination may increase desire to achieve forms of social capital associated with thinness to reduce future discrimination in other domains. They also point to Meyer's (2003) minority stress model, which links the stress associated with experiences of discrimination with higher levels of psychological disturbance. Individuals may internalize discriminatory experiences, which may result in negative self‐evaluations (Hatzenbuehler 2009) and, in turn, may prompt disordered eating (Raffoul et al. 2023).

Extended versions of Objectification Theory may help explain the relationship between perceived discrimination and eating disorder symptoms. Objectification Theory posits that objectifying experiences can lead to internalization of appearance ideals, self‐surveillance, and body shame, which in turn increase the risk of disordered eating (Fredrickson and Roberts 1997). To enhance its relevance for minoritized populations, Moradi (2010) extended the framework to include racial discrimination, conceptualizing discriminatory experiences as sociocultural appearance‐based stressors that reinforce dominant appearance ideals and self‐objectification. In support of this, research with Asian American women has demonstrated associations between racism and disordered eating via self‐objectification pathways (Cheng et al. 2017). Although originally developed to explain women's experiences, evidence suggests that self‐objectification may also contribute to disordered eating among men (Schaefer and Thompson 2018). This may be informative given emerging evidence of elevated eating disorder symptoms among men in several Asian contexts (Chua et al. 2021, 2022).

Colorism is discrimination based on a skin shade hierarchy whereby people with darker skin are penalized while those with lighter skin are privileged (Dixon and Telles 2017). Colorism exists globally, occurring both within and between ethnic/racialized groups (Dixon and Telles 2017). Like other forms of discrimination, colorism can operate at a systemic level (e.g., via media and advertising), within institutions (e.g., schools, healthcare settings, courts), interpersonally (e.g., between individuals), and can be internalized at the individual level (Strmic‐Pawl et al. 2021). Although people of varying skin shades can experience and be affected by colorism, those with dark skin tend to report more frequent instances of colorism (Craddock, Phoenix, et al. 2023; Craddock, Gentili, et al. 2023). Research shows that people with dark skin face harsher school discipline, longer prison sentences for the same crime, and economic penalties when compared with peers with lighter skin shades (Crutchfield et al. 2022; Sissoko et al. 2023; Yetsenga et al. 2024).

Colorism is widespread across Asia (Bettache 2020; Dixon and Telles 2017). Light or “Asian white skin” is central to a pan‐Asian appearance ideal, symbolizing wealth, social status, femininity, and beauty (Yip et al. 2019, 74). Although the origins of colorism, shaped by unique histories of colonization, class hierarchies, modernization, and globalization, vary across Asia (Bettache 2020), popular media (e.g., Bollywood, K‐Pop, K‐Beauty, and Japanese Beauty) and celebrity culture privilege light skin and frequently reproduce colorist stereotypes in the region (Yip et al. 2019). Moreover, the profitable and expanding skin‐lightening industry further reflects and reinforces the prejudice in the region (Pollock et al. 2021; Yip et al. 2019). Drawing on the extended conceptualization of Objectification Theory detailed above, colorism may operate as an appearance‐based form of discrimination that reinforces dominant appearance ideals and heightens self‐objectification processes, increasing vulnerability to disordered eating.

Few studies have examined the specific role of colorism on eating disorder risk factors, behaviors, and diagnoses. Several UK‐based studies have found associations between experiences of colorism and worse body image and lower self‐esteem in minoritized ethnic (mostly Asian and Black) adolescents and adults (Craddock, Phoenix, et al. 2023; Craddock, Gentili, et al. 2023; Craddock et al. 2025). Next, based on nationally representative US data, Beccia et al. (2020) found that Latinas who reported experiences of colorism were nearly nine times more likely to report lifetime binge eating than those reporting no discrimination. In another large probability sample of Black American adults, Oh et al. (2021) found self‐reported experiences of colorism from other Black people were positively associated with over 20% greater odds of having lifetime anorexia or bulimia. Therefore, given the relevance of colorism across Asia and the rising incidence of eating disorders in East and Southeast Asia, research examining colorism and eating disorder symptoms in this region is needed.

### 1.1 The Present Study

In response to calls for more research on colorism in Asia (Bettache 2020), the primary aim of this study was to explore the relationship between experiences of colorism (operationalised as skin shade discrimination) and disordered weight control behaviors (DWCBs) among women and men in Hong Kong, Malaysia, Singapore, and Thailand. Drawing on an extended version of Objectification Theory (Fredrickson and Roberts 1997), we hypothesized that experiencing any skin shade discrimination would be positively associated with DWCBs in women and men. We also hypothesized that a greater number of skin shade discrimination experiences would be associated with elevated DWCB risk. That is, there would be an association by breadth of reported experiences based on the cumulative number of different examples of skin shade discrimination an individual experienced.

The secondary aim was to test whether skin shade, gender, age, and country/region independently moderated the relationship between experiencing any skin shade discrimination and DWCBs. We hypothesized that self‐reported skin shade would moderate this relationship, such that it would be stronger among participants with darker skin compared with those with lighter skin since we expected that those with darker skin would experience more skin shade discrimination. We anticipated that the relationship between skin shade discrimination and DWCBs would be stronger in women compared with men due to the disproportionate burden of colorism on women (Hunter 2002; Reece 2021). We did not make a priori directional hypotheses for the moderation by age or country/region but given the nascency of research on this topic and in this world region, we included both as exploratory analyses.

## 2 Method

### 2.1 Procedure

Participants completed a single online survey as part of a larger investigation of body image and disordered eating among adults in Hong Kong, Malaysia, Singapore, and Thailand. Data collection was designed using a nonprobability, targeted sampling method (with quotas to approximate population demographics), and was conducted by YouGov, a market research and data analytics company. Data collection took place in September 2020 during the COVID‐19 pandemic. Each country/region was in a partial lockdown at the time of data collection. A detailed account of study data collection, sample selection, and survey translation is provided elsewhere (Chua et al. 2023). All participants in Thailand completed the survey in Thai. The majority (96.4%) of participants in Hong Kong completed the survey in traditional Chinese, and 45.6% of participants in Malaysia completed the survey in Bahasa Malaysia. The remaining participants in Hong Kong, Malaysia, and all participants in Singapore completed the survey in English. The project received Institutional Review Board (IRB) approval from Nanyang Technological University, Singapore.

### 2.2 Measures

#### 2.2.1 Explanatory Variable: Experiences of Skin Shade Discrimination

Interpersonal experiences of skin shade discrimination were assessed using an adapted version of the Everyday Discrimination Scale (EDS; Williams et al. 1997), a nine‐item self‐report measure of routine unfair treatment. The nine original discrimination scenarios (e.g., treated with less courtesy, treated with less respect, receiving poorer service, being perceived as unintelligent, inferior, dishonest, or threatening, being insulted, or harassed) were retained, but items were explicitly anchored to discrimination based on skin shade. Participants indicated whether each experience had occurred because of their skin shade during the past 3 months. The timeframe was shortened from the original measure to reduce recall bias and capture more proximal exposures likely to be relevant to current behaviors.

Responses were coded dichotomously (experienced vs. not experienced) for each item. A binary indicator of any skin shade discrimination exposure (any vs. none) was created for primary analyses to distinguish participants with recent experiences from those without exposure. In addition, a count score representing the number of different types of discriminatory experiences endorsed (range 0–9) was calculated to examine breadth of exposure across domains. This operationalization reflects the diversity of experiences rather than their frequency or cumulative intensity and therefore should not be interpreted as a direct measure of “dose.” Dichotomization and item counting involved a trade‐off between measurement precision and feasibility in a large multi‐country survey, reducing participant burden and emphasizing recent, recurrent experiences while limiting the ability to assess gradients of exposure.

The adapted scale demonstrated acceptable internal consistency in the present sample (Cronbach's *α* = 0.750), supporting reliability of the modified measure. Retention of the original item content and structure supports face validity of the adapted measure. However, the adaptation may not capture the full frequency or severity of discriminatory experiences.

#### 2.2.2 Outcome Variable: Disordered Weight Control Behaviors

DWCBs were conceptualized as a range of unhealthy or extreme behaviors undertaken with the intention of weight loss or weight management that may reflect varying clinical severity. DWCBs were assessed by presenting participants with nine behaviors aimed at controlling weight or body shape (vomiting, excessive exercising, fasting, use of laxatives, use of weight‐loss pills, use of fat burners, use of detox/cleansing dietary supplements, use of muscle‐building/sport‐performance dietary supplements, use of anabolic steroids). Excessive exercise was defined as “pushed yourself very hard; had to stick to a specific exercise schedule no matter what–for example even when you were sick/injured or if it meant missing a class or other important obligation; felt compelled to exercise”; and fasting was defined as “intentionally not eaten anything at all for at least 24 hours in an attempt to prevent weight gain (e.g., that is feared as a result of binge eating) or to lose weight.” Participants were asked to indicate if they had engaged in any of the behaviors in the past 3 months (“Yes” or “No”). A binary variable was created for any past 3‐month DWCBs (any/none).

#### 2.2.3 Covariates

Covariates included self‐reported demographic characteristics: marital status (married, in a relationship but not married, single), education level (below degree level, at least undergraduate degree level), employment status (full‐time, part‐time, full‐time student, other) and monthly household income level (low, low‐average, high‐average, high). Income was categorized according to the national monthly median (Hong Kong, Malaysia, and Singapore) or average (Thailand) household income per capita in line with the World Health Organization World Mental Health Survey.

#### 2.2.4 Moderators

Self‐reported skin shade was assessed using a purpose‐built skin shade color chart, which included 10 color options using the Pantone SkinTone Guide, with 1 being the lightest skin shade and 10 being the darkest. Skin shade, Age (years), Gender (woman, man), Geographic Region (Hong Kong, Malaysia, Singapore, Thailand) were all hypothesized to moderate the relationship between skin shade discrimination and DWCBs.

#### 2.2.5 Statistical Analyses

Multivariable binary logistic models were used to investigate whether experiences of skin shade discrimination in the past 3 months (binary predictor: no/yes) were associated with DWCBs in the same period (binary outcome: no/yes), and whether this association was moderated by self‐reported skin shade, age, gender, or country/region of origin, after controlling for relevant covariates. A hierarchical modeling approach was employed to test hypotheses, with all analyses conducted using SPSS version 29. Three hierarchically structured models were used. Model fit was evaluated using Akaike Information Criterion (AIC), Bayesian Information Criterion (BIC), and Nagelkerke *R* ^2^ (N‐*R* ^2^). Lower AIC and BIC values indicate better model fit, with BIC imposing a stronger penalty for model complexity than AIC, while N‐*R* ^2^ provides an estimate of explained variance. To aid interpretation, we additionally report the Number Needed to Take (NNT) as a complementary effect‐size metric for the association between skin shade discrimination and DWCBs. In this context, the NNT represents the number of individuals with recent experiences of skin shade discrimination who would need to be identified (relative to those without such experiences) to observe one additional case of DWCBs (Kraemer 2010). By way of example, an NNT of 5 indicates that, for every five individuals who reported recent experiences of skin shade discrimination, one additional case of DWCBs would be expected compared with five individuals who did not report experiences of skin shade discrimination. As a sensitivity analysis, the total number of endorsed DWCBs was also modeled as a count outcome using negative binomial regression; results are reported in the Supporting Information.

#### 2.2.6 Model 1: Association Between Colorism and DWCBs (Adjusted for Covariates)

The initial model included experiences of skin shade discrimination (shorthand = colorism) as the predictor, with marital status (three levels), educational attainment (two levels), employment status (four levels), and income status (four levels) as covariates. Odds ratios (ORs) with 95% confidence intervals (CIs) were used to quantify the adjusted association of skin shade discrimination with DWCBs.

#### 2.2.7 Model 2: Association Between Colorism and DWCBs, After Accounting for Demographic Factors

Model 2 includes main effects of potential moderators: skin shade (10‐point ordinal scale), age, gender (binary: man [0], woman [1]), and country/region (four levels). This model assessed whether including these variables improved model fit and estimated the relationships between experiences of skin shade discrimination and DWCBs after controlling for potential confounders and moderators.

#### 2.2.8 Model 3: Testing Differences in Associations Across Groups

Model 3 extends Model 2 by including two‐way interactions between skin shade discrimination and each potential moderator (colorism × skin shade; colorism × age; colorism × gender; colorism × country/region).

The statistical assumptions of the logistic models were evaluated. The Hosmer–Lemeshow test was used to assess lack of fit, indicating no significant discrepancies between observed and predicted probabilities (*p* > 0.05). Residual analysis was conducted to evaluate model specification, confirming the appropriateness of included predictors and functional form. Multicollinearity was assessed using variance inflation factors (VIFs), with all values below 2.5, suggesting no substantial multicollinearity.

Models were fitted with and without the variable income status (the only variable to have any missing values—9.2% of cases) using all available cases. Income status was not significantly associated with DWCB in any model (*p* > 0.05), and its inclusion or exclusion did not alter statistical conclusions. Therefore, results are reported for models excluding the variable income status. Skin shade was modeled both as a scale variable and as a 10‐point ordinal factor (nine degrees of freedom). Statistical conclusions were invariant to the specification of skin shade.

## 3 Results

Participants were 5251 adults (52.4% women) from Hong Kong (*n* = 1014), Malaysia (*n* = 1141), Singapore (*n* = 1049), and Thailand (*n* = 2047). Demographic characteristics are shown in Table 1.

**TABLE 1:** Participant demographics.

	Total	Hong Kong	Malaysia	Singapore	Thailand
	*n* = 5251	*n* = 1014	*n* = 1141	*n* = 1049	*n* = 2047
Gender (%)					
Women	52.5	48.1	58.1	50.4	52.5
Age group (%)					
18–24	17.0	9.8	28.7	7.6	18.9
25–34	27.3	17.6	35.5	19.6	31.4
35–44	21.7	18.9	19.9	20.9	24.4
45–54	16.4	21.3	10.3	19.4	15.8
55+	17.7	32.4	5.6	32.5	9.5
Ethnicity (%)					
Chinese	—	—	24.6	73.6	—
Malay	—	—	69.9	13.6	—
Indian	—	—	4.4	9.2	—
Other	—	—	1.1	3.3	—
Marital status (%)					
Married	43.6	57.0	39.2	58.9	31.6
In a relationship	10.4	9.3	6.9	7.2	14.5
Single/divorced widowed/separated	46.0	33.7	53.9	33.8	53.9
Education (%)					
Below university degree	47.6	49.5	55.3	51.4	40.4
University degree and above	52.4	50.5	44.7	48.6	59.6
Employment status (%)					
Working full‐time	59.1	70.8	48.8	61.5	59.1
Working part‐time	12.8	9.4	13.8	13.1	12.8
Full‐time student	8.7	5.1	16.3	8.4	8.7
Others	19.4	14.7	21.0	17.0	19.4
Household income (%)					
Low	32.9	19.8	35.5	38.2	35.3
Low‐average	27.3	29.1	22.8	27.6	28.8
High‐average	20.9	31.6	15.4	17.7	20.3
High	9.7	11.8	5.3	4.2	13.8
*Missing*	*9.2*	*7.7*	*21.0*	*12.3*	*1.8*

*Note:* Ethnicity data were not collected in Hong Kong or Thailand. National statistics indicate that the population of Hong Kong is 92% Chinese (2016 by‐census) and that Thailand is 75% Thai (YWAM Thailand). According to the Department of Statistics Singapore (2020), the population of Singapore is 74.4% Chinese, 13.4% Malays, and 9.0% Indians (9.0%). Values presented in italic font represent missing data.

Approximately one in four (25.3%) of respondents reported experiencing skin shade discrimination in the past 3 months (Hong Kong = 14.2%; Malaysia = 28.7%; Singapore = 21.3%; Thailand = 31.0%). There were no differences by gender except in Singapore, where men were more likely to report skin shade discrimination compared with women, 26.2% versus 16.4%, $\chi^{2}$ (df = 1) = 14.76, *p* < 0.001. The distribution of discriminatory experiences of colorism by gender within each country/region are presented in the Supporting Information (Table S1).

Approximately half (53.8%) of the total sample reported engaging in at least one DWCB in the past 3 months (Hong Kong = 40.5%; Malaysia = 56.9%; Singapore = 43.1%; Thailand = 64.2%). The percentage of participants who reported DWCBs in the past 3 months varied by gender in Hong Kong and in Thailand, but not in Malaysia or Singapore. In Hong Kong, women were more likely to report DWCBs in the past 3 months compared with men, 45.9% versus 35.6%, $\chi^{2}\;\left(\mathrm{df}=1\right)$ = 11.25, *p* < 0.001. Likewise, in Thailand, women were more likely to report DWCBs in the past 3 months compared with men, 67.1% vs. 61.0%, $\chi^{2}$ (df = 1) = 8.23, *p* = 0.004. The distribution of each disordered weight‐control behavior by gender for each country/region is mentioned in Supporting Information (Table S2).

To examine the robustness of findings across alternative operationalizations of DWCBs, we conducted supplementary analyses modeling the number of endorsed DWCBs as a count outcome using negative binomial regression. Overall, conclusions were largely consistent with those from the binary DWCB models. In models adjusting for study covariates and potential moderators, experiences of skin shade discrimination were associated with a higher number of DWCBs, and moderation patterns did not differ meaningfully from the primary analyses. Detailed results are presented in Supporting Information Table S3. One small difference emerged, such that being in a relationship (relative to being married) was associated with a lower number of endorsed DWCBs; however, this did not alter the pattern or interpretation of associations between skin shade discrimination and DWCBs.

Given the high proportions of DWCBs, we explored the past 3‐month frequency excluding use of (i) detox/cleansing dietary supplements and (ii) muscle‐building/sport‐performance dietary supplements as these are widely used and may be perceived as more benign than some of the other DWCBs (Ganson and Nagata 2024). However, we found the prevalence remained substantial even when excluding these two items, with rates only dropping slightly to 46.5% (Hong Kong = 35.7%; Malaysia = 51.1%; Singapore = 37.5%; Thailand = 54.0%), suggesting the finding is not solely driven by more normative behaviors. Further, the same patterns by gender and country/region were observed as above when just these two items were excluded. Therefore, we retained all nine DWCBs in the main analysis.

### 3.1 Associations Between Colorism and DWCBs

Model 1 is presented in Table 2. After controlling for study covariates (relationship status, employment status, and education), individuals experiencing skin shade discrimination in the past 3 months had more than five times the odds of engaging in DWCBs in the previous 3 months, Adjusted Odds Ratio (AOR) = 5.32, 95% CI [4.56, 6.19], NNT = 3. Notably, those educated to at least degree level were 1.3 times more likely to engage in DWCBs, AOR = 1.33, 95% CI [1.18, 1.48].

**TABLE 2:** Model 1: Association between experiences of skin shade discrimination and DWCBs (adjusted for covariates).

	*p*	Odds ratio	95% CI
Relationship status (overall test)	0.205	—	—
In a relationship	0.152	1.159	0.947, 1.419
Single	0.145	1.099	0.968, 1.248
Employment status (overall test)	< 0.001	—	—
Working part time	0.321	0.913	0.764, 1.093
Full‐time student	0.306	0.890	0.713, 1.112
Other	< 0.001	0.690	0.593, 0.803
University degree and above	< 0.001	1.329	1.180, 1.485
Colorism	< 0.001	5.315	4.562, 6.191
Constant	< 0.001	0.721	—

*Note:* Reference category for Marital Status is married; Reference category for Employment Status is working full‐time. Model 1. AIC = 6626.60, BIC = 6679.13, N‐*R* ^2^ = 0.15.

Model 2 and Model 3 are presented in Table 3. Model fit improved markedly from Model 1 to Model 2, with substantial reductions in AIC and BIC and increased N‐*R* ^2^. Inclusion of interaction terms in Model 3 resulted in a slight additional improvement in AIC and N‐*R* ^2^ but a higher BIC, indicating modest gains in explanatory power relative to increased complexity. Given the study's a priori hypotheses regarding moderation, Model 3 was retained as the primary model despite the higher BIC. Model 2 shows that after controlling for study covariates and potential moderators (skin shade, age, gender, and country/region), those experiencing skin shade discrimination in the past 3 months were still close to five times more likely to engage in DWCBs in the previous 3 months (AOR = 4.76, 95% CI [4.07, 5.57]), NNT = 3. Educational attainment at the degree level or above remained significant (*p* = 0.003). Skin shade was not significantly associated with DWCBs. However, being a woman was associated with elevated odds of DWCBs (AOR = 1.24, 95% CI [1.10, 1.40]) and the prevalence of DWCB decreased with increasing age (*p* < 0.001). There were also statistically significant differences between country/region (*p* < 0.001) with DWCBs being more prevalent in Malaysia and in Thailand than in Hong Kong, consistent with unadjusted analyses.

**TABLE 3:** Model 2: Association between experiences of skin shade discrimination and DWCBs after accounting for demographic factors and Model 3: Testing differences in association between experiences of skin shade discrimination and DWCBs across groups.

	Model 2			Model 3		
	*p*	Odds ratio	95% CI	*p*	Odds ratio	95% CI
Relationship status	0.017	—	—	0.014	—	—
In a relationship	0.093	0.83	0.67, 1.03	0.111	0.84	0.68, 1.04
Single	0.005	0.82	0.71, 0.94	0.004	0.81	0.71, 0.94
Employment status	< 0.001	—	—	< 0.001	—	—
Working part time	0.159	0.88	0.73, 1.05	0.164	0.88	0.73, 1.06
Full‐time student	0.002	0.69	0.55, 0.88	0.003	0.71	0.56, 0.89
Other	< 0.001	0.65	0.56, 0.76	< 0.001	0.65	0.55, 0.76
University degree and above	0.003	1.20	1.06, 1.36	0.004	1.20	1.06, 1.36
Colorism	< 0.001	4.76	4.07, 5.57	< 0.001	5.15	2.29, 11.60
Skin shade	0.558	0.99	0.97, 1.02	0.494	0.99	0.96, 1.02
Age	< 0.001	0.99	0.98, 0.99	< 0.001	0.99	0.98, 0.99
Woman	< 0.001	1.24	1.10, 1.40	< 0.001	1.33	1.16, 1.52
Country	< 0.001	—	—	< 0.001	—	—
Malaysia	< 0.001	1.56	1.29, 1.90	< 0.001	1.73	1.40, 2.14
Singapore	0.726	1.03	0.86, 1.25	0.176	1.15	0.94, 1.41
Thailand	< 0.001	2.17	1.83, 2.58	< 0.001	2.23	1.85, 2.69
Colorism × demographic						
Colorism × age	—	—	—	0.044	1.01	1.00, 1.03
Colorism × gender	—	—	—	0.010	0.66	0.48, 0.91
Colorism × shade	—	—	—	0.559	1.02	0.96, 1.09
Colorism by country				0.008	—	—
Colorism × Malaysia	—	—	—	0.022	0.515	0.292, 0.909
Colorism × Singapore	—	—	—	0.004	0.439	0.251, 0.768
Colorism × Thailand	—	—	—	0.268	0.739	0.432, 1.263
Constant	0.969	1.007	—	0.85	0.99	—

*Note:* Reference category for marital status is married; reference category for employment status is working full‐time; reference category for country/region is Hong Kong. Model 2: AIC = 6459.92, BIC = 6545.01, N‐*R* ^2^ = 0.19. Model 3: AIC = 6455.50, BIC = 6579.00, N‐*R* ^2^ = 0.20.

**FIGURE 1:**
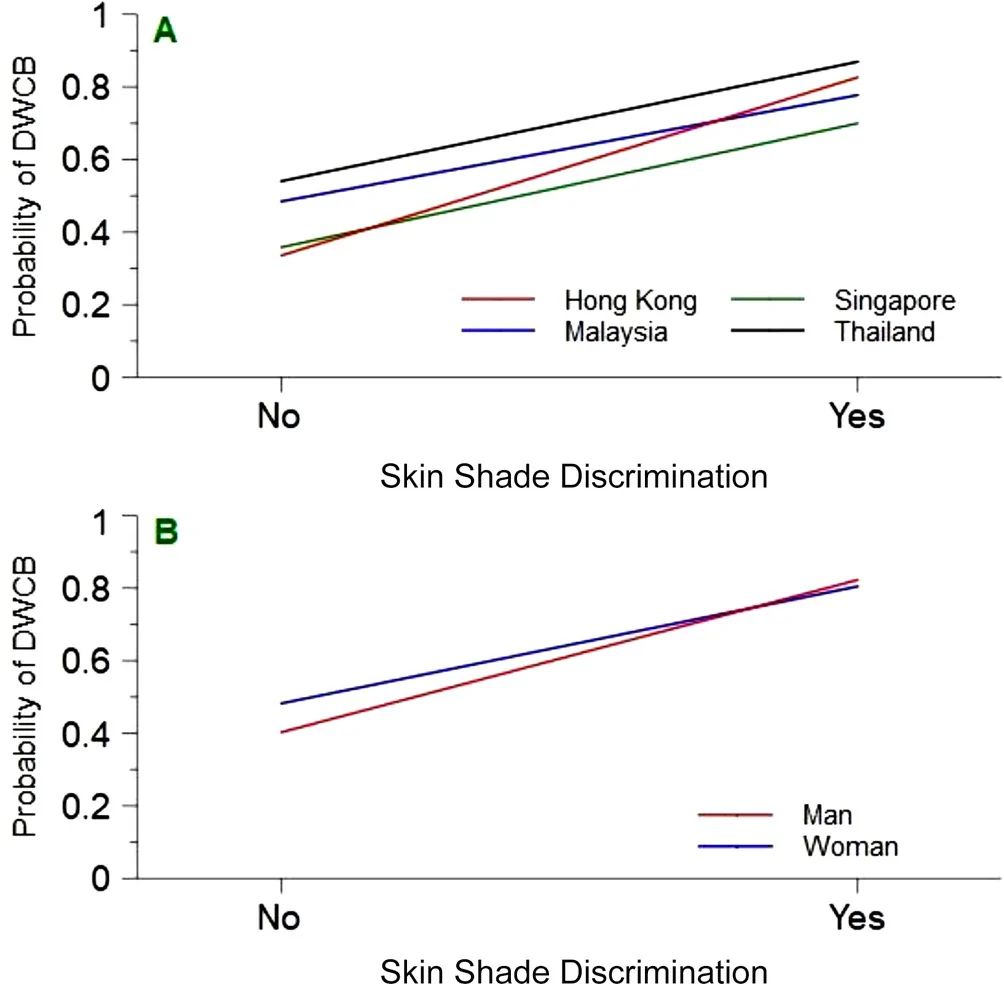
Logistic model (Model 3) probability of a DWCB for country (A) and gender (B) by experiences of skin shade discrimination.

**FIGURE 2:**
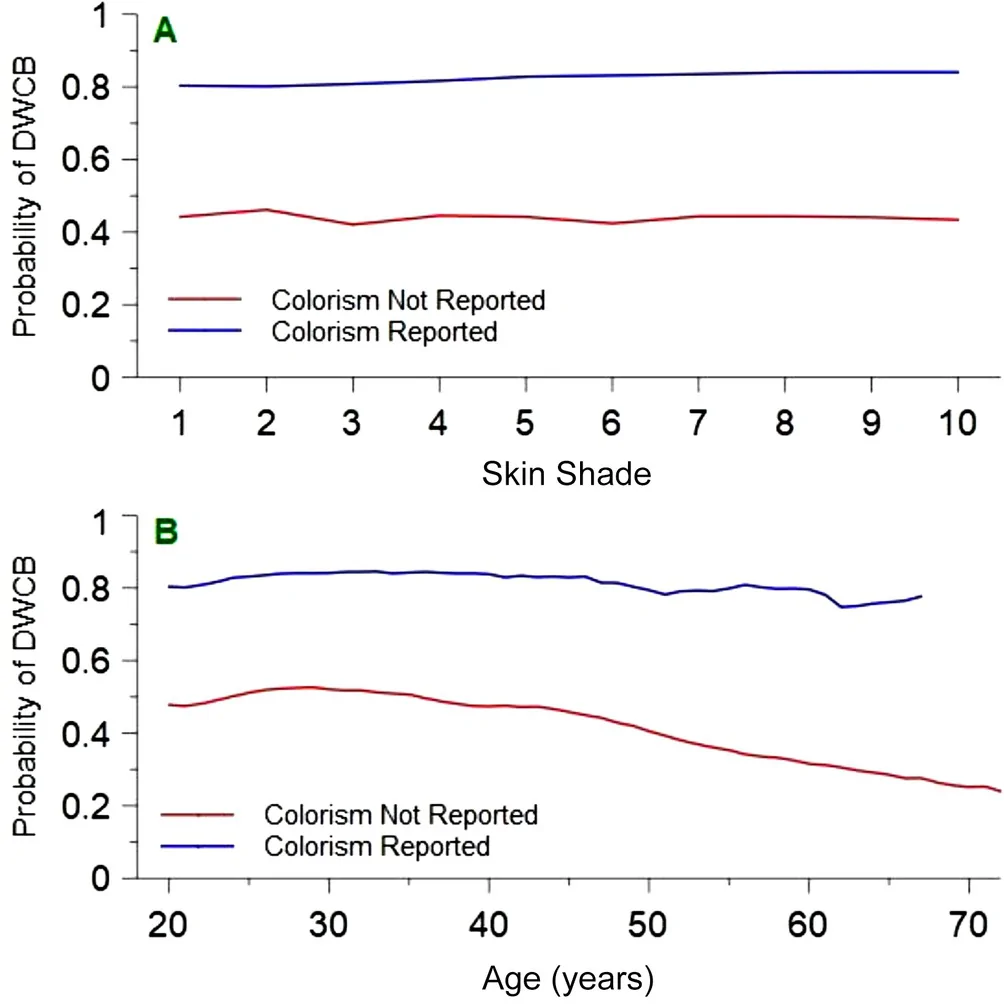
Logistic model (Model 3) probability of a DWCB for self‐reported skin shade (A) and age (B) by experiences of skin shade discrimination (colorism).

In Model 3, the overall NNT for skin shade discrimination was approximately 3, although significant interactions indicate modest variation by region and gender. Model 3 and Figure 1A show that the relationship between skin shade discrimination and DWCBs was moderated by country/region (*p* = 0.008). Among participants not reporting skin shade discrimination, the probability of DWCBs was relatively lower and varied across countries/regions, whereas among those reporting skin shade discrimination, the probability of DWCBs was higher with little variation between countries/regions. Model 3 and Figure 1B illustrate moderation by gender (*p* = 0.010). Among participants not reporting skin shade discrimination, women had a higher probability of DWCBs than men; however, among those reporting skin shade discrimination, the probability of DWCBs was similarly elevated for women and men. Skin shade was not significantly related to DWCBs and did not moderate the relationship between skin shade discrimination and DWCBs (Figure 2A). Age was significantly related to DWCBs and modestly moderated the association between skin shade discrimination and DWCBs (*p* = 0.044), particularly at older ages (Figure 2B).

### 3.2 Association Between DWCBs and Breadth of Colorism Experiences

**FIGURE 3:**
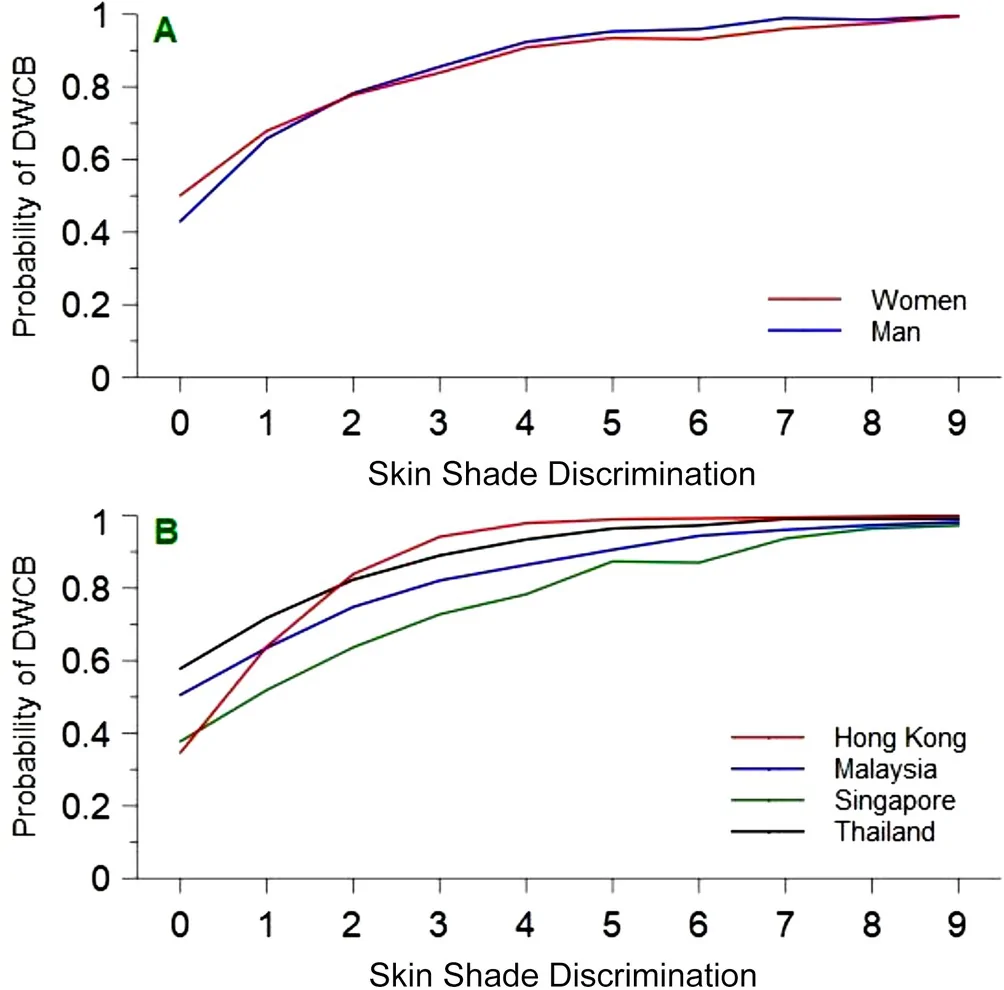
Logistic probability of a DWCB for gender (A) and country/region (B) by number of reported skin shade discrimination (i.e., colorist) events.

Additional exploratory analyses indicated that a broader range of reported skin shade discrimination experiences was associated with increased odds of engaging in DWCBs in the past 3 months (*p* < 0.001). All statistical conclusions reported in Model 3 remain when the occurrence of any skin shade discrimination is replaced by the number of reported skin shade discrimination events except for the age‐by‐number of reported skin shade discrimination interaction, which no longer achieved statistical significance (*p* = 0.254). Of note, both gender (*p* = 0.026) and country/region (*p* = 0.002) moderated this positive association. However, the difference between women and men, although statistically significant (*p* = 0.026) as shown in Figure 3A, was modest. Differences in the odds of a DWCB between countries/region are quite pronounced at low counts of skin shade discrimination, and these differences quickly diminish with increasing counts as shown in Figure 3B.

## 4 Discussion

This study examined the association between recent experiences of colorism and DWCBs among adults in four Asian countries/regions. Experiences of skin shade discrimination were associated with nearly fivefold higher odds of reporting DWCBs within the same 3‐month period, even after accounting for demographic factors. This finding aligns with a growing body of research linking appearance‐based discrimination to adverse mental health outcomes and maladaptive coping, including disordered eating. While causal inference is not possible due to the cross‐sectional design, the use of the same recall period supports comparability between measures.

The association between skin shade discrimination and DWCBs should be understood within the broader context of the well‐documented adverse effects of colorism on health. Prior research links experiences of colorism to elevated risk of psychological distress, poor body image, diminished self‐esteem, worse physical health, and a diagnosis of a psychiatric disorder (Craddock, Phoenix, et al. 2023; Craddock, Gentili, et al. 2023; Monk 2021; Oh et al. 2021). From this perspective, eating disorder risk does not represent an isolated consequence of colorism but rather one component of a wider constellation of negative health impacts. Situating disordered eating within this broader pattern highlights the public health significance of colorism and underscores the need for prevention efforts that address colorism as a structural and sociocultural determinant of health.

### 4.1 Main Findings: Colorism and DWCBs

Participants who reported experiences of skin shade discrimination in the past 3 months were approximately five times more likely to report DWCBs compared with those who reported not experiencing any prejudice or discrimination based on their skin shade. An NNT of 3 further indicates that the effects are not trivial. These findings corroborate prior research demonstrating associations between colorism and disordered eating or eating disorders among Latina women living in the United States (Beccia et al. 2020; Velez et al. 2015) and Black American women and men (Oh et al. 2021). The results also indicate that a greater range of reported colorism experiences was associated with increased risk of DWCBs. However, these associations should be interpreted cautiously. Engagement in DWCBs may also shape how individuals perceive, interpret, or recall experiences of colorism, and both may be influenced by unmeasured factors such as psychological distress, coping styles, or broader social stressors. Together, these findings underscore the need for more in‐depth research to understand the mechanisms underlying the relationship between colorism and disordered eating across diverse groups using qualitative and longitudinal methods.

### 4.2 Hypothesized and Exploratory Moderators

Contrary to our hypothesis, skin shade did not moderate the relationship between self‐reported experiences of skin shade discrimination and DWCBs. The fact that skin shade did not modify findings suggests that experiences of colorism were associated with an increased risk of DWCBs regardless of self‐reported skin shade. This supports understandings that perceived experiences of discrimination can be a more useful indicator on wellbeing outcomes than individual characteristics subject to prejudice (Pascoe and Smart Richman 2009; Schmitt et al. 2014). Conversely, these results may be an artifact of variation by country/region with different racialized groups. For example, those who are ethnically Thai likely have darker skin than those who are Chinese, living either in Hong Kong, Singapore, or Malaysia.

Our exploratory analysis showed country/region moderated the relationship between skin shade discrimination and DWCBs. Specifically, the relationship between skin shade discrimination and DWCBs appeared stronger in the Hong Kong sample compared with the samples from Malaysia, Singapore, and Thailand. Given that participants in Hong Kong reported the lowest rate of experiences of colorism in this study (14% compared with 21%–31%) it is possible that individuals living in Hong Kong who experienced skin shade prejudice may have felt more isolated. In turn, they may have felt a greater drive to seek a sense of control of how they are perceived and treated by others by pursuing DWCBs (Mason et al. 2021). However, more in‐depth qualitative and longitudinal research is needed to test this explanation.

We found that DWCBs were more prevalent in Malaysia and Thailand than in Hong Kong. This should be interpreted with caution given limited epidemiological data in the region and the fact that DWCBs do not equate to DSM‐5‐TR eating disorder diagnoses. These behaviors may instead reflect sociocultural and public health influences distinct from those associated with clinical disorders. For example, several Southeast Asian countries are undergoing rapid nutritional transitions characterized by shifts toward energy‐dense diets and rising average weight (Tee and Voon 2024). In response, governments have implemented strong public health initiatives emphasizing weight management and lifestyle modification, which may inadvertently normalize dieting and other weight‐control practices (Tee and Voon 2024). In Malaysia, religious and voluntary fasting practices which may, at times, be framed as strategies for weight control may also shape attitudes toward restrictive eating (Abdullah et al. 2024). Further research is needed to clarify how sociocultural, religious, and structural factors contribute to cross‐national differences in disordered weight‐control behaviors.

Consistent with our hypotheses, gender moderated the relationship between skin shade discrimination and DWCBs, such that the association was stronger for women than for men. Societal appearance ideals emphasize thinness and light skin (among other traits) as markers of feminine beauty resulting in women often facing greater societal pressure to be thin and to have light skin in many Asian populations (Yip et al. 2019). In turn, experiences of colorism may lead women to engage more frequently in weight control behaviors to obtain or maintain one form of “body capital” aligned with these ideals (Mason et al. 2021). It would be informative to explore the role of perceived control of weight/body shape and skin shade as a possible explanation for this relationship. An important caveat would be to closely examine such relationships by region or country context, as for example, some research in China has found no differences between women and men on numerous body image outcomes (Jackson and Chen 2015).

Finally, exploratory findings indicated that age marginally moderated the relationship between experiences of skin shade discrimination and DWCBs though this was most relevant to the older age range. Specifically, for people who reported skin shade discrimination, their probability of DWCBs remained constant by age while for those who reported not experiencing colorism, the probability of DWCBs declined after the age of 50 years (approximately). Meta‐analysis evidence suggests that DWCBs decline from adolescence through to midlife (Romano et al. 2020) though less is known about older adults. It is possible that experiencing discrimination may be a driver for DWCBs in later life. It is also plausible that there could be an underlying cumulative effect whereby adults in their 50s, 60s, and 70s who reported experiencing colorism in the past 3 months have experienced colorism over many decades and have adopted various coping mechanisms or strategies to control their appearance in other ways. This highlights a need for more in‐depth research among older adults who are engaging in DWCBs to more thoroughly explain motivations and driving influences.

### 4.3 Limitations

The following limitations are acknowledged. First, the cross‐sectional design precludes causal inference, and the observed associations may reflect more complex or bidirectional processes. Second, data were collected during the COVID‐19 pandemic, a period associated with elevated disordered eating behaviors (Schneider et al. 2023); replication under non‐pandemic conditions would help determine the stability of these findings. Third, the online survey format may have excluded individuals not proficient in written Bahasa Malaysia, English, Thai, or traditional Chinese, as well as those without regular internet access, thereby limiting generalizability.

Fourth, several relevant variables were not assessed. We did not measure experiences of racism alongside experiences of colorism, limiting our ability to disentangle the specific effects of colorism from broader racialized discrimination. This omission may be particularly important in Malaysia and Singapore, where multiple racialized groups coexist within distinct sociopolitical contexts, which influence how different racialized groups are treated (Chong Ching Yee and Jones 2025; Loh 2025). Additionally, racialized group data were not collected in Hong Kong and Thailand; although these settings are often described as relatively homogeneous, this limits the precision with which colorism experiences can be situated within broader social hierarchies. Height and weight were not collected, precluding calculation of body mass index (BMI) and examination of its potential associations with study variables, including whether weight status confounded relationships with DWCBs.

Finally, our modification of the EDS and dichotomization of DWCBs involved a trade‐off between measurement precision and feasibility in a large multi‐country survey. Collapsing response options reduced participant burden and potential recall bias by emphasizing recent, recurrent experiences, but resulted in loss of information regarding frequency and severity, limiting assessment of dose–response relationships. Although the large sample size provided adequate statistical power to detect non‐trivial effects, the measures should be interpreted as indicators of meaningful recent experiences of discrimination rather than the full spectrum of discrimination intensity. Therefore, the findings should not be interpreted as evidence regarding the severity or clinical classification of eating disorders.

### 4.4 Future Research

The current study prompts several avenues for future research. First, extending this work to additional Asian contexts would strengthen understanding of the generalizability of these findings. For example, given the salience of colorism in the Indian subcontinent and diaspora (Bajwa et al. 2023; Mishra et al. 2023), examining these associations in South Asian populations would be a valuable next step. Second, qualitative research could deepen understanding of the mechanisms linking experiences of colorism to DWCBs. In‐depth qualitative approaches focusing on diverse demographic and intersectional profiles (e.g., young Indian men in Malaysia, migrant workers from Indonesia living in Singapore) could provide nuanced insight into how colorism is experienced and how it may shape weight control practices within specific social contexts. Relatedly, longitudinal designs will be valuable for clarifying the temporal ordering of these associations and for testing more complex models that incorporate potential bidirectional effects, mediating processes, and contextual influences.

Third, future work could examine whether colorism experienced from racialized peers or from an outgroup alters the magnitude of the relationship between self‐reported experiences of colorism and DWCBs. Emerging research in the United Kingdom and United States suggests that mental health outcomes, including eating disorder symptoms, may differ depending on whether colorism is experienced within or across racialized groups (Craddock, Phoenix, et al. 2023; Oh et al. 2021). Expanding this line of research to multiethnic societies such as Malaysia and Singapore^1^ could yield useful insights.

Fourth, future research should examine a broader range of disordered eating behaviors, including binge eating, to provide a more complete understanding of how colorism relates to eating disorder risk. Using culturally appropriate, multidimensional, validated measures of disordered eating would enable more clinically meaningful interpretation and allow examination of whether colorism is differentially associated with milder versus more severe forms of pathology. Additionally, incorporating measures of mood, depression, and psychological distress could clarify potential pathways linking experiences of colorism to disordered eating, including possible mediating or moderating effects. Fifth, it would be valuable to investigate the impact of macro‐level and institutional colorism on eating disorder symptoms in order to gain a more complete understanding of how skin shade discrimination operates across levels of influence. Given that many corporations profit from promoting unrealistic appearance ideals and marketing skin‐lightening products, examining the role of industries and social media represents an important macro‐level direction. Similarly, research on institutional colorism and its relationship to eating disorder symptoms and mental health more broadly could inform policy and structural interventions. Finally, given the observed association between experiences of colorism and DWCBs, future research should examine protective factors that may attenuate this relationship. Identifying individual, interpersonal, and community‐level buffers would directly inform eating disorder prevention strategies and clinical practice in East and Southeast Asia.

### 4.5 Conclusion

The current study found that experiencing colorism, operationalised here as skin shade discrimination, was associated with DWCBs in women and men living in Hong Kong, Malaysia, Singapore, and Thailand. Consequently, eating disorder prevention and treatment in East and Southeast Asia should consider the role of colorism as a potential correlate or contributing factor of disordered eating alongside established sociocultural risk factors such as weight stigma and the promotion of thin/lean body shape ideals. Future research should further explore the role of colorism on DWCBs in different Asian countries and examine the role of colorism from different sources (e.g., ingroup and outgroup) and different societal levels (e.g., systemic and institutional colorism). Both longitudinal and qualitative methods will be valuable in this effort.

## Author Contributions

**Nadia Craddock:** conceptualization, writing – original draft, methodology, writing – review and editing, project administration, resources. **S. Bryn Austin:** funding acquisition, writing – review and editing, methodology, project administration. **Flora Or:** resources, writing – reviewing and editing. **Wipada Rodtanaporn:** resources, writing – review and editing. **Paul White:** data curation, formal analysis, methodology, writing – original draft, writing – review and editing. **Sook Ning Chua:** methodology, writing – review and editing, project administration, resources, investigation, data curation.

## Funding

Funding for data collection was supported by the Strategic Training Initiative for the Prevention of Eating Disorders.

## Ethics Statement

Ethical approval for data collection was granted by the Institutional Review Board at Nanyang Technological University, Singapore. We have followed all relevant ethical guidelines for human subjects research.

## Conflicts of Interest

The authors declare no conflicts of interest.

## Supporting information


**Table S1:** Percentage of skin shade discriminatory behaviors experienced by gender within each country.
**Table S2:** Percentage of specific disordered weight control behaviors by gender within each country.
**Table S3:** Incidence rate ratios from negative binomial models predicting number of disordered weight control behaviors.

## Data Availability

Data will be made available on request.

## Endnotes

^1^
Both Malaysia and Singapore have three main racialized groups. The largest racialized groups in Malaysia—Bumiputera, including Malay and indigenous people (62.5%), Chinese (20.6%) and Indian (6.2%). The largest racialized groups in Singapore—Chinese (74%), Malay (13%), and Indian (9%) (OOSGA, 2023).
