# Supplementary material for: Skin Shade Discrimination Is Associated With Disordered Weight Control Behaviors in Hong Kong, Malaysia, Singapore, and Thailand
Source: Int J Eat Disord. 2026 Apr 30;59(9):1945–56. doi: 10.1002/eat.70117 (PMC13536177; doi:10.1002/eat.70117)
Supplement: Supplementary file 1 [file EAT-59-1945-s001.docx]

Experiences of colorism are associated with disordered weight control behaviors in Hong Kong, Malaysia, Singapore, and Thailand

Nadia Craddock, S Bryn Austin, Flora Or, Wipada Rodtanaporn, Paul White, Sook Ning Chua

**Supplementary Online Files**

**Table S1.**

Percentage of skin shade discriminatory behaviors experienced by gender within each country

|  |  | Hong Kong | Malaysia | Singapore | Thailand | Total |
| --- | --- | --- | --- | --- | --- | --- |
| Act as if better | Male | 5.1 | 12.8 | 10.6 | 10.4 | 9.8 |
| than you | Female | 4.1 | 12.7 | 6.8 | 12.7 | 10.0 |
|  |  |  |  |  |  |  |
| Not treated | Male | 4.0 | 8.6 | 7.9 | 8.9 | 7.6 |
| with respect | Female | 5.7 | 9.5 | 5.7 | 8.2 | 7.6 |
|  |  |  |  |  |  |  |
| People act as | Male | 3.6 | 9.2 | 8.5 | 8.7 | 7.7 |
| if you are not | Female | 3.5 | 9.4 | 5.3 | 8.8 | 7.3 |
| smart |  |  |  |  |  |  |
| Not treated | Male | 4.0 | 7.9 | 6.7 | 9.0 | 7.3 |
| with courtesy | Female | 4.1 | 7.8 | 5.9 | 8.8 | 7.2 |
|  |  |  |  |  |  |  |
| Being called | Male | 3.0 | 7.5 | 4.4 | 8.1 | 6.2 |
| names or | Female | 3.3 | 7.8 | 2.1 | 8.3 | 6.1 |
| insulted |  |  |  |  |  |  |
| Received poorer | Male | 2.3 | 3.2 | 7.5 | 5.2 | 5.0 |
| service | Female | 4.1 | 4.6 | 5.3 | 6.2 | 5.4 |
|  |  |  |  |  |  |  |
| People act as if | Male | 3.2 | 7.5 | 6.3 | 6.6 | 6.0 |
| you are dishonest | Female | 3.3 | 5.6 | 1.9 | 5.7 | 4.5 |
|  |  |  |  |  |  |  |
| People act as if | Male | 2.3 | 5.2 | 4.6 | 7.0 | 5.2 |
| afraid of you | Female | 3.1 | 3.8 | 2.6 | 4.8 | 3.8 |
|  |  |  |  |  |  |  |
| Harassed or | Male | 1.5 | 3.1 | 4.4 | 4.7 | 3.7 |
| threatened | Female | 3.3 | 2.4 | 2.6 | 3.5 | 3.1 |
|  |  |  |  |  |  |  |
| Sample size:  Female 2754 (Hong Kong 488, Malaysia 663, Singapore 529, Thailand 1074)  Male 2497 (Hong Kong 526, Malaysia 478, Singapore 520, Thailand 973) | | | | | | |

**Table S2.**

Percentage of specific disordered weight control behaviors by gender within each country.

|  |  | Hong Kong | Malaysia | Singapore | Thailand | Total |
| --- | --- | --- | --- | --- | --- | --- |
| Excessive | Men | 16.7 | 25.9 | 20.8 | 26.6 | 23.2 |
| Exercise | Women | 16.8 | 21.6 | 12.1 | 19.6 | 18.1 |
|  |  |  |  |  |  |  |
| Fasted | Men | 10.1 | 26.2 | 13.1 | 13.8 | 15.2 |
| (>= 24 hours) | Women | 9.2 | 24.4 | 11.5 | 16.6 | 16.2 |
|  |  |  |  |  |  |  |
| Detox | Men | 4.2 | 14.0 | 9.4 | 17.1 | 12.2 |
| Supplements | Women | 13.9 | 19.3 | 12.5 | 24.2 | 19.0 |
|  |  |  |  |  |  |  |
| Muscle | Men | 6.1 | 11.7 | 9.8 | 19.6 | 13.2 |
| Builders | Women | 5.5 | 5.7 | 3.0 | 13.8 | 8.3 |
|  |  |  |  |  |  |  |
| Fat | Men | 4.4 | 8.4 | 5.2 | 12.2 | 8.4 |
| Burners | Women | 6.6 | 8.9 | 4.3 | 20.2 | 12.0 |
|  |  |  |  |  |  |  |
| Laxatives | Men | 1.1 | 4.2 | 3.8 | 7.7 | 4.8 |
|  | Women | 3.7 | 5.0 | 2.6 | 11.9 | 7.0 |
|  |  |  |  |  |  |  |
| Weight Loss | Men | 3.0 | 4.0 | 2.9 | 5.0 | 4.0 |
| Pills | Women | 3.9 | 6.0 | 3.6 | 7.0 | 5.6 |
|  |  |  |  |  |  |  |
| Anabolic | Men | 1.3 | 1.9 | 1.9 | 7.7 | 4.0 |
| Steroids | Women | 1.0 | 1.8 | 0.4 | 5.0 | 2.7 |
|  |  |  |  |  |  |  |
| Self-induced | Men | 0.8 | 3.3 | 2.9 | 3.1 | 2.6 |
| Vomit | Women | 2.3 | 4.5 | 1.9 | 3.6 | 3.3 |
|  |  |  |  |  |  |  |
| Sample size:  Women 2754 (Hong Kong 488, Malaysia 663, Singapore 529, Thailand 1074)  Men 2497 (Hong Kong 526, Malaysia 478, Singapore 520, Thailand 973) | | | | | | |

**Table S3.**

Incidence Rate Ratios from Negative Binomial Models Predicting Number of Disordered Weight Control Behaviours

|  | *Model S1* | | | | | | *Model S2* | | | | |  |
| --- | --- | --- | --- | --- | --- | --- | --- | --- | --- | --- | --- | --- |
|  | *p* | | IRR | | 95% CI | | *p* | | Odds ratio | 95% CI | |  |
| **Relationship Status** | <.001 | | - | | - | | <.001 | |  |  | |  |
| In a relationship | .004 | | 0.81 | | 0.70, 0.93 | | .012 | | 0.79 | 0.66, 0.95 | |  |
| Single | <.001 | | 0.83 | | 0.75, 0.91 | | .018 | | 0.87 | 0.77, 0.98 | |  |
| **Employment Status** | <.001 | | - | | - | | <.001 | |  |  | |  |
| Working part time | .207 | | 0.92 | | 0.81, 1.04 | | .633 | | 0.96 | 0.82, 1.12 | |  |
| Full time student | <.001 | | 0.72 | | 0.61, 0.84 | | .002 | | 0.73 | 0.59, 0.89 | |  |
| Other | <.001 | | 0.76 | | 0.67, 0.85 | | <.001 | | 0.79 | 0.69, 0.90 | |  |
| **University degree and above** | .007 | | 1.12 | | 1.03, 1.22 | | .103 | | 1.09 | 0.98, 1.21 | |  |
| **Colorism** | <.001 | | 2.24 | | 2.05, 2.44 | | .003 | | 2.20 | 1.32, 3.66 | |  |
| **Skin Shade** | .262 | | 0.99 | | 0.97, 1.01 | | .163 | | 0.98 | 0.96. 1.01 | |  |
| **Age** | <.001 | | 0.99 | | 0.98, 0.99 | | <.001 | | 0.99 | 0.98, 0.99 | |  |
| **Woman** | .007 | | 1.12 | | 1.03, 1.22 | | .003 | | 1.17 | 1.05, 1.30 | |  |
| **Country** | <.001 | | - | | - | | <.001 | |  |  | |  |
| Malaysia | <.001 | | 1.45 | | 1.26, 1.66 | | <.001 | | 1.53 | 1.29, 1.81 | |  |
| Singapore | .273 | | 1.08 | | 0.94, 1.25 | | .299 | | 1.09 | 0.92, 1.29 | |  |
| Thailand | <.001 | | 1.72 | | 1.52, 1.95 | | <.001 | | 1.88 | 1.62, 2.17 | |  |
| **Colorism x Demographic** |  | |  | |  | | .218 | |  |  | |  |
| Colorism x Age | - | | - | | - | | .061 | | 1.01 | 1.00, 1.02 | |  |
| Colorism x Gender | - | | - | | - | | .109 | | 0.87 | 0.73, 1.04 | |  |
| Colorism x Shade | - | | - | | - | | .406 | | 1.02 | 0.98, 1.05 | |  |
| **Colorism by Country** |  | |  | |  | | .117 | |  |  | |  |
| Colorism x Malaysia | | - | | - | |  | | .165 | 0.80 | | 0.59, 1.10 | |
| Colorism x Singapore | | - | | - | |  | | .456 | 0.89 | | 0.64, 1.22 | |
| Colorism x Thailand | | - | | - | |  | | .050 | 0.75 | | 0.57, 1.00 | |
| **Constant** | .492 | | 0.92 | | 0.73, 1.17 | | .614 | | 0.94 | 0.70, 1.24 | |  |
